# Cadaveric Study on the Anatomical Variations in the Branching Pattern of the Tibial Nerve to the Deep Muscles of the Posterior Compartment of the Leg

**DOI:** 10.7759/cureus.90631

**Published:** 2025-08-20

**Authors:** Dimple Dev V, Suman U

**Affiliations:** 1 Department of Anatomy, Amrita School of Medicine, Amrita Institute of Medical Science, Amrita Vishwa Vidyapeetham, Kochi, IND; 2 Department of Anatomy, Kempegowda Institute of Medical Sciences, Bengaluru, IND

**Keywords:** anatomical variation, cadaveric investigation, flexor muscles, motor innervation, nerve branching pattern, posterior leg, tibial nerve

## Abstract

Objectives: This study aimed to classify and assess the motor branching patterns of the tibial nerve (TN) to the flexor digitorum longus (FDL), tibialis posterior (TP), and flexor hallucis longus (FHL) muscles.

Methodology: This observational cadaveric study examined the motor branching patterns of the TN by dissecting 60 adult lower limbs preserved in formalin. Standard dissection techniques exposed the TN from its origin at the apex of the popliteal fossa, where it arises from the sciatic nerve, to its bifurcation into the medial and lateral plantar nerves within the tarsal tunnel near the medial malleolus, with particular attention paid to its branches supplying the FHL, FDL, and TP muscles.

Results: Three distinct branching patterns were identified based on the number and configuration of motor branches supplying the posterior compartments’ deep muscles. Type I was observed in 42 (70%) limbs and exhibited individual branches to each FHL, FDL, and TP muscle. Type II, found in 14 (23.33%) limbs, demonstrated two main branches supplying all three muscles. Type III, seen in four (6.67%) limbs, showed a single common branch innervating all three muscles. Representative dissection photographs were obtained to illustrate each branching pattern.

Conclusion: This study revealed three distinct TN branching patterns to the deep posterior compartment of the leg, with the most common type being the TN giving separate branches to each muscle. These findings have significant clinical relevance for improving the safety of surgical procedures, regional anesthesia, and the management of TN pathologies. A comprehensive understanding of these variations is essential for minimizing the risk of nerve injury and guiding more effective surgical and therapeutic strategies.

## Introduction

The tibial nerve (TN), a significant branch of the sciatic nerve (SN), is essential for innervating the posterior compartment of the leg, particularly the deep muscles responsible for plantar flexion of the foot and flexion of the toes. After passing through the popliteal fossa, it enters the posterior aspect of the leg and gives off multiple muscular branches along its course. A detailed understanding of its branching pattern to the deep muscles of the leg is crucial for clinical applications such as nerve blocks, managing entrapment syndromes, and reconstructive surgeries. According to Desai and Cohen-Levy [1], the TN supplies motor innervation to key muscles, including the tibialis posterior (TP), flexor digitorum longus (FDL), and flexor hallucis longus (FHL), highlighting its importance in lower limb function [1].

The TN divides into articular, cutaneous, and muscular branches in the popliteal fossa. The medial inferior genicular nerve's articular branch supplies the medial aspect of the knee joint capsule. The medial sural cutaneous nerve typically descends between the two heads of the gastrocnemius, piercing the crural fascia near the distal part of the popliteal fossa [2]. Muscular branches in this region innervate the medial head (MH) and lateral gastrocnemius head (LH), soleus (So), plantaris (Pl), and popliteus (Po) muscles [3]. Although these branches are frequently described in anatomical and surgical literature, considerable variability in their configuration has been documented by multiple authors [4]. The underlying causes of this variation remain unclear. Prior studies have particularly highlighted variations in the innervation of the gastrocnemius heads and soleus, often neglecting detailed descriptions of the branches to the Pl and Po muscles [5-7]. Clarifying the organization and relationships of these muscular branches is key to understanding their embryological origins [7].

Additionally, ongoing debate surrounds the developmental origin of the plantaris muscle, specifically, whether it arises from the deep muscular anlage of the leg or a more proximal region involving the tibial side of the LH and So [8-10]. While these muscles are not the primary focus of this study, mapping the TN’s motor branches to the deep posterior compartment may offer anatomical clues relevant to unresolved questions of regional muscle development. Identifying consistent branching patterns could aid in refining criteria for classifying muscle groups and contribute to a clearer understanding of lower limb morphogenesis [11].

Although previous anatomical studies have described the TN’s course and motor branches, there is no explicit agreement on the precise branching pattern in the popliteal fossa and leg, particularly regarding its innervation of the deep posterior compartment muscles. Specifically, limited data exist on the branching patterns of the TP, FDL, and FHL, including their orientation, branching patterns, and insertion points. The lack of a consistent framework in existing literature challenges clinical applications and developmental understanding. This study aims to fill that gap by systematically analyzing the TN’s motor branches to TP, FDL, and FHL in human cadaveric limbs.

## Materials and methods

This study examined 60 formalin-preserved adult cadaveric lower limbs, irrespective of sex or laterality, obtained from the Department of Anatomy at Kempegowda Institute of Medical Sciences in Bengaluru, India. Specimens with visible deformities, trauma, or prior surgical interventions were excluded.

Dissection protocol

Dissections followed standard procedures outlined in Cunningham’s Manual of Practical Anatomy, 15^th^ edition, volume 1 [12]. Each limb was positioned prone with the hip and knee fully extended. A midline incision was made from the posterior aspect of the thigh through the popliteal fossa down to the ankle. After skin reflection, the deep crural fascia was carefully removed to expose the underlying muscles. The triceps surae was excised at its origin, and the intermuscular septum was incised longitudinally to reveal neurovascular structures. The TN was traced from its origin at the SN in the popliteal fossa to its terminal bifurcation into the medial and lateral plantar nerves, ensuring preservation of the epineurium and avoiding mechanical disruption or displacement of nerve branches. 

Assessment of TN branching

The study focused on documenting motor branches of the TN, including their number and insertion points into the FHL, FDL, and TP muscles. The nerve was traced distally until its bifurcation into medial and lateral plantar nerves, with care taken to prevent artificial separation.

Data collection and analysis

To improve visualization during dissection, the tibial nerve (TN) was painted with a yellow paint. High-resolution photographs were taken at multiple stages to document the branching patterns. These photographic records were systematically cross-checked with the dissection notes to ensure accuracy in the identification and labeling of branches. Branching types were classified, and the findings were summarized as frequencies and percentages (N (%).

## Results

The study was conducted on 60 lower limbs of adult human cadavers preserved in formalin. The parameters relevant to the objectives were carefully observed, recorded, and organized in Table 1.

**Table 1:** Distribution of tibial nerve (TN) branching patterns to the deep muscles of the leg's posterior compartment (N = 60) Type I: Three separate motor branches arising from the TN, each independently innervating the flexor hallucis longus (FHL), flexor digitorum longus (FDL), and tibialis posterior (TP) muscles. Type II: Two main motor branches from the TN, each giving rise to further branches supplying all three deep muscles of the leg. Type III: A single common motor branch arising from the TN that subsequently divides to innervate the FHL, FDL, and TP muscles.

Type of specimen	Total number N (%)
Type I	42 (70%)
Type II	14 (23.33%)
Type III	4 (6.67%)

**Figure 1:**
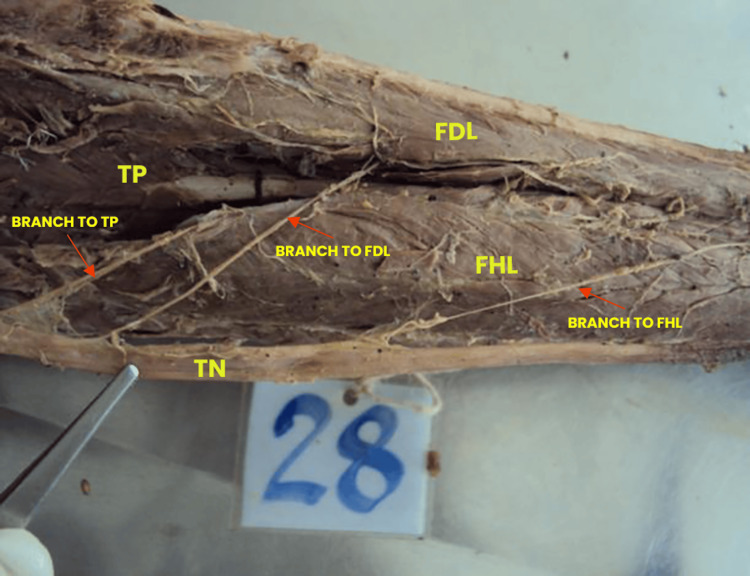
Type I branching pattern of the TN The left limb was oriented in a prone position with the hip and knee fully extended to expose the posterior compartment for tibial nerve (TN) dissection. The TN was found to give rise to three distinct motor branches, each independently innervating the flexor hallucis longus (FHL), flexor digitorum longus (FDL), and tibialis posterior (TP) muscles.

The branching pattern of the TN in the posterior crural region was classified into three distinct types based on the number and distribution of motor branches innervating the deep posterior compartment muscles. Type I was observed in 42 (70%) limbs, in which the TN gave rise to separate branches for each of the deep posterior compartment muscles: FHL, FDL, and TP (Table 1, Figure 1).

**Figure 2:**
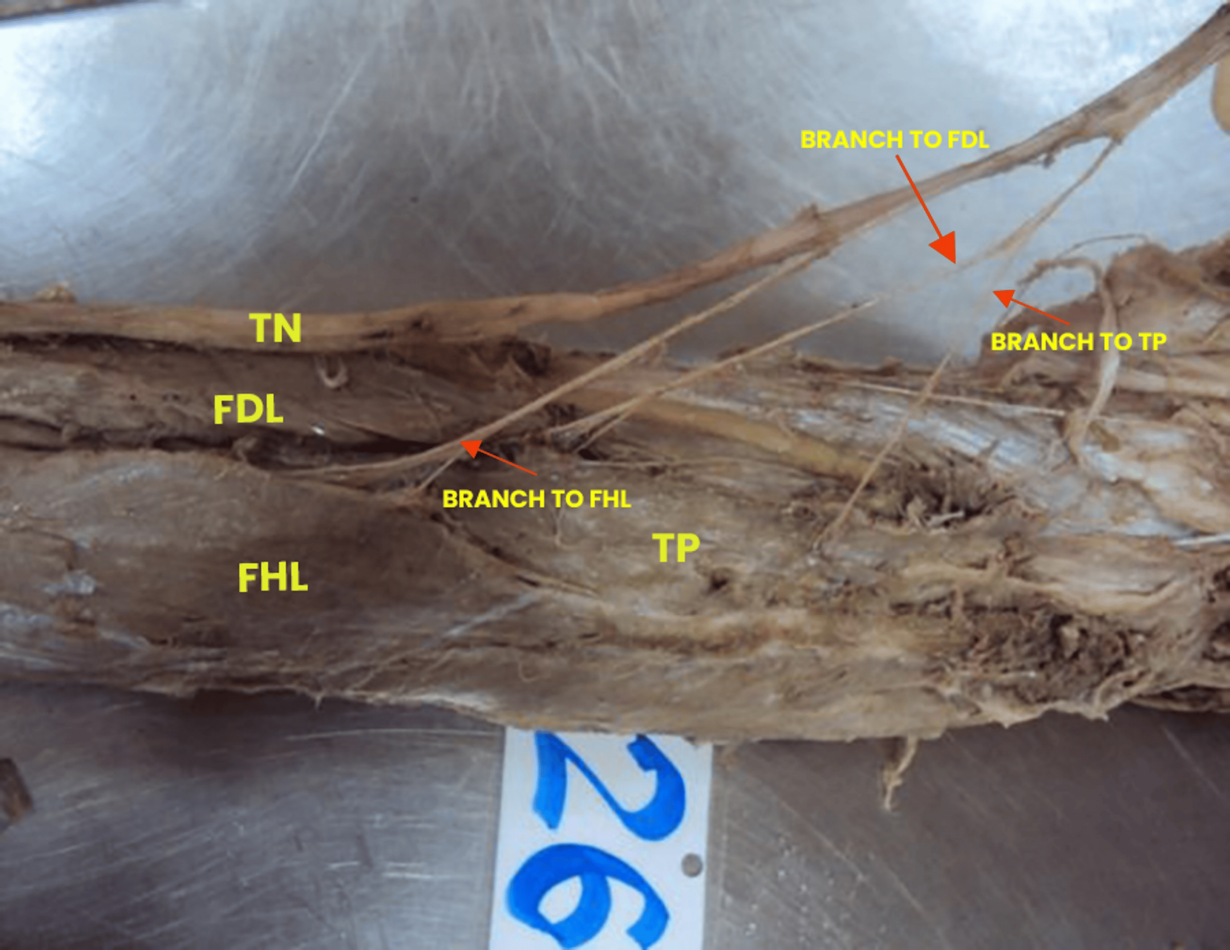
Type II branching pattern of the TN The tibial nerve (TN) bifurcates into two primary motor branches, which further subdivide to innervate the flexor hallucis longus (FHL), flexor digitorum longus (FDL), and tibialis posterior (TP) muscles. One branch supplies the TP and FDL in this configuration, while the other innervates the FHL.

Type II was identified in 14 (23.33%) limbs, where the TN was divided into two main branches, each giving off further motor branches to the deep muscles (Table 1, Figure 2).

**Figure 3:**
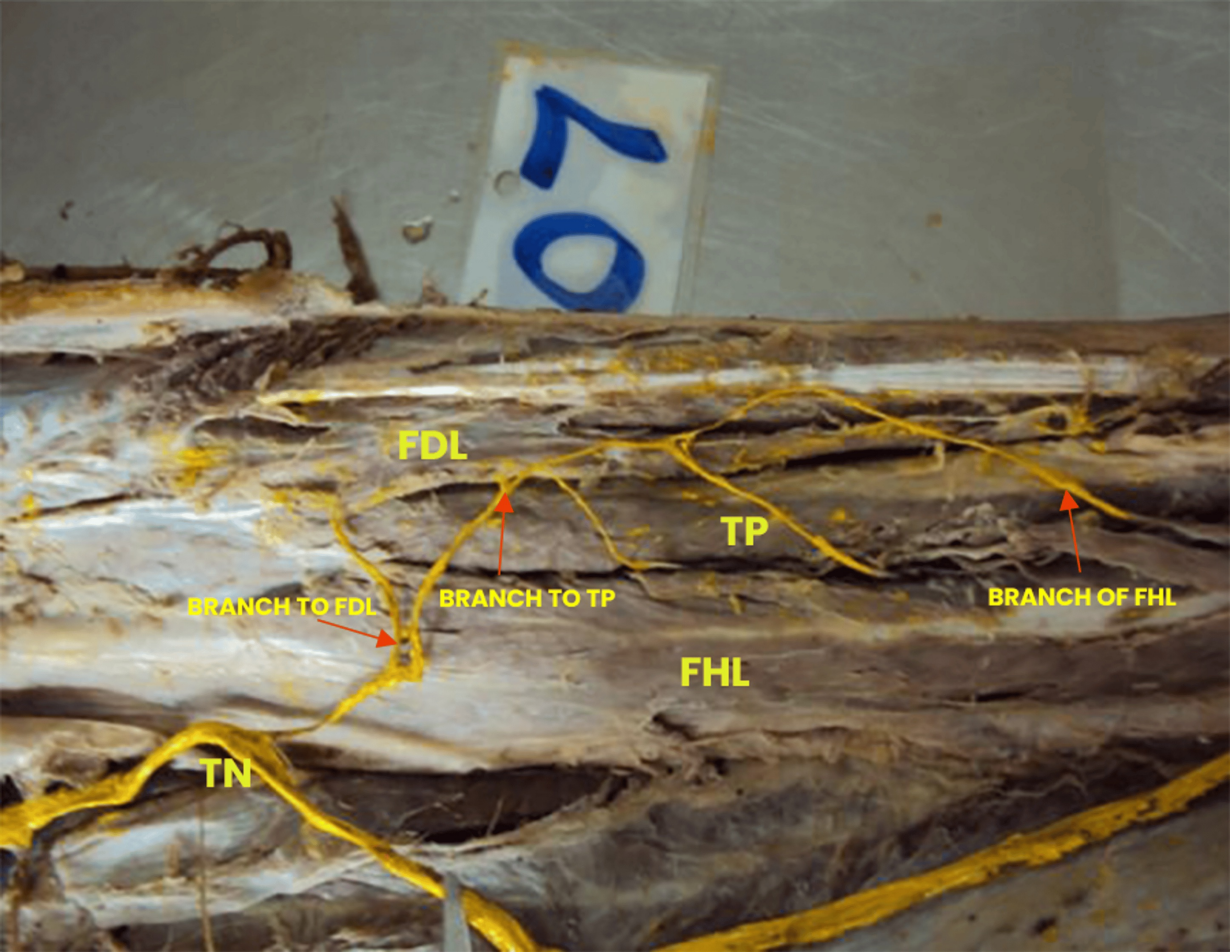
Type III branching pattern of the TN A single motor branch arises from the tibial nerve (TN). Subsequently, it divides into smaller branches that collectively innervate the flexor hallucis longus (FHL), flexor digitorum longus (FDL), and tibialis posterior (TP) muscles.

Type III was identified in four limbs (6.67%), characterized by the presence of a single, thick motor branch arising from the TN. This common trunk coursed anteromedially and entered the posterior compartment at a slightly oblique angle. After traveling a short distance, it gave rise to three sequential muscular branches. The first branch coursed medially to innervate the TP, followed by a second branch that ascended to supply the FDL. The third and final branch extended deeper and slightly laterally to reach the FHL. This unified branching arrangement diverged from the typical individual origin pattern and was consistent across the four limbs exhibiting this type. The anatomical orientation is illustrated in Figure 3, and a summary is provided in Table 1.

## Discussion

Understanding the branching patterns of the TN is essential for various clinical interventions, including posterior compartment of leg decompression surgeries, precise nerve blocks, tarsal tunnel syndrome management, and foot drop treatment. To the best of our knowledge, this is the first study to classify the motor branches of the TN to the TP, FDL, and FHL muscles in the posterior leg, thereby addressing a significant gap in the existing literature. To our knowledge, only one previous study, by Andreasen Struijk et al. [13], has presented findings relevant to all three muscles concurrently; however, their emphasis was on fascicular mapping within the tarsal tunnel rather than proximal branching patterns, which are the focus of the present work. Our study fills this gap by mapping and categorizing motor branches in the posterior crural region into three types: Type I (70%), with independent branches to each muscle; Type II (23.33%), with two main branches of the TN giving rise to smaller motor branches to supply the deep muscles of the back of the leg; and Type III (6.67%), with one main branch giving rise to separate motor branches to each muscle of the back of the leg. In Type I, the TN gives rise to three distinct motor branches, each independently innervating the TP, FDL, and FHL muscles. This pattern confirms the anatomical independence of motor innervation and represents the most common branching configuration. Its surgical relevance lies in the facilitation of precise nerve localization and preservation. Our classification aligns with findings by Andreasen Struijk et al. [13], who identified largely distinct fascicles within the tarsal tunnel, supporting the concept of separate motor pathways. Although earlier works (e.g., Louisia and Masquelet [14], Dellon and Mackinnon [15], Havel [16], Davis and Schon [17]) focused on TN bifurcation, they did not explicitly delineate motor-branch independence. Our findings demonstrate that such independent branching is frequently established proximal to the tunnel, extending previous tunnel-focused observations. Apaydin et al. [18] also reported a similar triple-branch frequency using bony landmarks, reinforcing the consistency of Type I patterns.

The predominance of Type I supports prior anatomical principles discussed by Andreasen Struijk et al. [13], who emphasized the importance of fascicular specificity in designing functional electrical stimulation (FES) systems. Their observation of distinguishable fascicles (noted in 80% of specimens) complements our findings of distinct proximal motor branches.

Type II is characterized by two primary motor branches arising from the TN: one innervating the FDL and TP, and the other supplying the FHL. This partial fusion of motor pathways may contribute to variability in clinical outcomes, particularly in nerve block efficacy and surgical intervention. While previous studies have alluded to shared trunks [12], few have explicitly described this specific branching pattern [14,15]. Our analysis quantifies and anatomically defines this two-branch configuration in the posterior crural region. Andreasen Struijk et al. [13] alluded to fascicular clustering that may account for such patterns, while Davis and Schon [17] and Dellon and Mackinnon [15] discussed anatomical variability without providing explicit quantification. Apaydin et al. [18] reported similar bifurcated trunk arrangements in approximately 30% of specimens, and Banik and Guria [19] offered corroborative evidence from distal tarsal tunnel dissections.

Our findings are also consistent with previously reported intratunnel bifurcation rates: 73% by Louisia and Masquelet [14], 95% by Dellon and Mackinnon [15], 93% by Havel et al. [16], and 90% by Davis and Schon [17]. Although we focused on the posterior crural region, all branching patterns (100%) were observed proximal to a consistently defined anatomical zone, corroborating and extending these distal observations.

In Type III, a single motor branch arises from the TN, dividing into fascicles supplying the TP, FDL, and FHL. This configuration is anatomically less standard and may be overlooked during surgical procedures, increasing the risk of simultaneous injury to all three muscles. This variant underscores the importance of detailed preoperative nerve mapping and meticulous dissection. While Kurtoglu et al. [20] discussed high divisions and accessory muscles, mentioning a unified trunk innervating all three muscles is rare. Though Andreasen Struijk et al. [13] noted closely apposed fascicles in some specimens, their focus remained on distal applications rather than surgical implications in the proximal posterior compartment of the leg. Our documentation of this unified trunk introduces clinically significant detail and highlights a potential source of combined motor loss during surgical intervention. Although Banik and Guria [19] described distal bifurcations suggestive of Type III-like variants, our identification of a single motor trunk to all three deep muscles appears novel and of direct surgical relevance.

Davis and Schon [17] suggested that atypical TN branching proximal to the flexor retinaculum could explain inconsistencies between clinical symptoms and electromyography (EMG) findings. Identifying Type II and III variants may help clarify such discrepancies, as these patterns involve motor branches arising from one or two standard trunks instead of directly from the TN.

The surgical implications are considerable. Andreasen Struijk et al. [13] and Dellon and Mackinnon [15] emphasized that unrecognized anatomical variability increases the risk of inadvertent nerve injury. In our study, 30% of specimens deviated from the conventional Type I pattern, underscoring the importance of recognizing these variations, particularly during decompression or soft tissue procedures.

From an anesthetic perspective, branching variability may affect the success of TN blocks, particularly in surgeries requiring targeted analgesia (e.g., hallux valgus correction or total knee arthroplasty) [21]. Burton et al. [22] reported that TN blocks are highly effective for postoperative analgesia; however, their success depends on the accurate localization of nerve branches. Recognizing more proximal or clustered branching patterns, as in our Type II and III variants, may improve injection site selection and enhance outcomes, especially in pediatric or minimally invasive contexts [23,24]. While TN blocks have shown efficacy in postoperative pain management [22], their success hinges on accurate localization of nerve branches. Recognizing clustered or proximal branching, as seen in our Type II and III patterns, may improve injection site selection and clinical outcomes. Ultrasonographic studies by Picelli et al. [25] support this, demonstrating the reliability of localizing the TP motor branch using consistent anatomical landmarks. Deltombe et al. [26] similarly emphasized selective targeting of motor branches for spasticity management.

Although Kurtoglu et al. [20] associated high TN division with accessory muscles, such as the accessory FDL, we did not identify similar correlations in our specimens. This may be due to sample variation or highlights the need for larger-scale studies to capture rare anatomical variants. Dellon [15] emphasized TN vulnerability during high tibial osteotomy and fascial release, concerns reinforced by our findings, particularly regarding Type II and III variants. Finally, the detailed anatomical mapping presented in this study may support future applications in harvesting vascularized nerve grafts, particularly in limb salvage procedures that require precise and individualized nerve planning [27].

Limitations of the study

This study has several limitations that should be considered when interpreting its findings. First, using formalin-fixed cadaveric specimens, while essential for structural preservation, may cause tissue shrinkage and altered nerve morphology, potentially affecting the accuracy of branching pattern assessments. Second, although the sample size (n = 60) is reasonably robust, it may still be insufficient to capture the full spectrum of rare anatomical variations, particularly in the less common Type II and Type III branching configurations. Third, demographic details such as age, sex, and medical history of the cadavers were not documented, limiting the ability to correlate branching patterns with biological or clinical variables.

Additionally, as this was a cross-sectional anatomical study, functional correlations, such as the clinical impact of these branching patterns on nerve conduction or surgical outcomes, could not be established. The findings may not be fully generalizable to live clinical settings, particularly in pathological changes, inflammation, or trauma cases. Finally, the study focused exclusively on the posterior compartment of the leg and did not explore potential associations with variations in surrounding musculature, vascular structures, or developmental anomalies.

Future studies with larger, more diverse sample sizes, supplemented by histological and functional analyses, are warranted to expand upon these anatomical insights and enhance their translational relevance in surgical and anesthetic practice.

## Conclusions

This study identified three distinct patterns of TN branches that innervate the deep muscles of the posterior compartment of the leg, with the predominant pattern involving individual branches to each muscle. These findings are essential for enhancing the safety and effectiveness of surgical procedures, regional anesthesia, and treating conditions affecting the TN. The typical branching pattern observed in this study supports previous anatomical research and contributes valuable data specific to the posterior compartment of the leg. Understanding these patterns can help reduce the risk of nerve injury and guide more informed surgical and therapeutic decisions.
